# Serum of Post-COVID-19 Syndrome Patients with or without ME/CFS Differentially Affects Endothelial Cell Function In Vitro

**DOI:** 10.3390/cells11152376

**Published:** 2022-08-02

**Authors:** Lavinia Flaskamp, Constanze Roubal, Steven Uddin, Franziska Sotzny, Claudia Kedor, Sandra Bauer, Carmen Scheibenbogen, Martina Seifert

**Affiliations:** 1 Institute for Medical Immunology, Charité-Universitätsmedizin Berlin, Corporate Member of Freie Universität Berlin and Humboldt Universität zu Berlin, 13353 Berlin, Germany; lavinia.flaskamp@med.lmu.de (L.F.); constanze.roubal@charite.de (C.R.); steven.uddin@charite.de (S.U.); franziska.sotzny@charite.de (F.S.); claudia.kedor@charite.de (C.K.); sandra.bauer@charite.de (S.B.); carmen.scheibenbogen@charite.de (C.S.); 2 BCRT-Berlin Institute of Health (BIH) Center for Regenerative Therapies, Charité-Universitätsmedizin Berlin, 10178 Berlin, Germany; 3 DZHK (German Center for Cardiovascular Research), Partner Site, 10785 Berlin, Germany

**Keywords:** endothelial cells, angiogenesis, endothelial dysfunction, post-COVID syndrome, myalgic encephalomyelitis/chronic fatigue syndrome, autoantibodies

## Abstract

A proportion of COVID-19 reconvalescent patients develop post-COVID-19 syndrome (PCS) including a subgroup fulfilling diagnostic criteria of Myalgic encephalomyelitis/Chronic Fatigue Syndrome (PCS/CFS). Recently, endothelial dysfunction (ED) has been demonstrated in these patients, but the mechanisms remain elusive. Therefore, we investigated the effects of patients’ sera on endothelia cells (ECs) in vitro. PCS (n = 17), PCS/CFS (n = 13), and healthy controls (HC, n = 14) were screened for serum anti-endothelial cell autoantibodies (AECAs) and dysregulated cytokines. Serum-treated ECs were analysed for the induction of activation markers and the release of small molecules by flow cytometry. Moreover, the angiogenic potential of sera was measured in a tube formation assay. While only marginal differences between patient groups were observed for serum cytokines, AECA binding to ECs was significantly increased in PCS/CFS patients. Surprisingly, PCS and PCS/CFS sera reduced surface levels of several EC activation markers. PCS sera enhanced the release of molecules associated with vascular remodelling and significantly promoted angiogenesis in vitro compared to the PCS/CFS and HC groups. Additionally, sera from both patient cohorts induced the release of molecules involved in inhibition of nitric oxide-mediated endothelial relaxation. Overall, PCS and PCS/CFS patients′ sera differed in their AECA content and their functional effects on ECs, i.e., secretion profiles and angiogenic potential. We hypothesise a pro-angiogenic effect of PCS sera as a compensatory mechanism to ED which is absent in PCS/CFS patients.

## 1. Introduction

Myalgic encephalomyelitis/chronic fatigue syndrome (ME/CFS) is a chronic and debilitating disease with exertion intolerance and persistent fatigue as symptomatic cornerstones [1]. Similarly, following mild to moderate COVID-19, a substantial number of patients have been reported to suffer from long-term health consequences closely resembling those described for ME/CFS including fatigue and chronic pain [2,3,4]. Despite an incompletely understood disease aetiology, the most frequently reported ME/CFS triggers are viral infections [5]. On this background, in our recent observational study we could indeed diagnose a subset of post-COVID-19 fatigue patients with ME/CFS [6]. 

While the complex nature of the disease has made elucidation of the underlying pathomechanisms difficult, there is ample evidence for disturbances in immune, metabolic and autonomic function [5,7]. Interestingly, those imbalances have also been linked to endothelial dysfunction (ED) [8,9,10], a disorder characterised by disturbed vascular endothelial cell (EC) responses. Those include among others orchestration of local vasodilation, prevention of inappropriate coagulation or maintenance of the endothelial barrier integrity [11]. Given the numerous roles of vascular ECs in physiology, ED unsurprisingly represents an independent cardiovascular risk factor. In ME/CFS, several clinical studies have provided evidence for ED playing a role in the disease [12,13,14] which could also be observed in a number of the abovementioned post-COVID-19 fatigue patients with or without ME/CFS [15]. Specifically, both patient groups displayed elevated levels of the ED biomarker endothelin-1 with a reduced hyperaemic index in some of the patients. In this context, it is also worth noting that SARS-CoV-2 is known to target the endothelium via the highly expressed ACE2 receptor, leading to ED and subsequently numerous cardiovascular complications [16]. Nevertheless, how ED develops and the importance of its contributions to clinical manifestations such as cerebral hypoperfusion or orthostatic intolerance in the patients remains unclear. Potential candidates contributing to ED may be autoimmune mediators including autoantibodies, cytokines or altered immune cell populations [17], as described in other immunological disorders such as systemic sclerosis (SSc) or systemic lupus erythematosus [18,19]. Similarly, autoantibodies against distinct target structures as well as dysregulated serum or plasma-derived cytokines have been reported in ME/CFS cohorts [5,20]. 

In our study, we sought to determine alterations in EC function in vitro following their exposure to post-COVID-19 syndrome (PCS) patient serum including individuals with or without ME/CFS, i.e., PCS/CFS and PCS, respectively, from the same cohort described by Kedor et al. and Haffke et al. [6,15]. With a particular focus on immune mediators, we analysed serum for putative molecules involved in EC damage including cytokines and anti-endothelial cell autoantibodies (AECAs). Specifically, changes in the EC secretion profile or inappropriate EC activation served as functional readouts which could unravel a novel contributing factor to the development of ED. Moreover, differences between the two patient groups may provide additional insights into the mechanistic divergence between ME/CFS and other post-viral fatigue conditions such as PCS. 

## 2. Methods

### 2.1. Patients

Overall, 30 post-COVID-19 patients (mild to moderate disease course) with persistent fatigue and exertion intolerance, for at least 6 months, were recruited within an ongoing observational study from November 2020 until February 2021. Previous SARS-CoV-2 infection was confirmed by PCR or serology (SARS-CoV-2 IgG/IgA). Patients were diagnosed by consultation at our outpatient clinic and presence as well as severity of symptoms were assessed based on the 2003 Canadian Consensus Criteria (CCC) by questionnaires [21]. PCS/CFS patients met the criteria for fatigue, post-exertional malaise (PEM), sleep dysfunction and pain, suffered at least from two neurological/cognitive manifestations and at least from one symptom of the two categories: autonomic, neuroendocrine and immune manifestations as defined in the CCC, with the exception, that in accordance with the studies of L. Jason and colleagues, a minimum of 14 h (instead of 24 h) of PEM was sufficient to meet criteria for PEM [22]. Disability was examined using the Bell score, fatigue using Chalder Fatigue Score (Table 1), according to which PCS patients experienced similarly severe impairments in their daily life, however, they did not fulfil the CCC requirements with regards to a minimum of 14 h PEM, as detailed in the articles published by Kedor et al. [6] and Haffke et al. [15]. Additionally, a number of PCS patients did not meet the CCC criteria for neurological/cognitive symptoms.

Pre-existing co-morbidities, including fatigue, served as exclusion criteria. 14 healthy age- and sex-matched subjects were recruited as a control group. All investigations using this patient cohort have been approved by the Ethics Committee of Charité Universitätsmedizin Berlin (EA2/066/20) in accordance with the 1964 Declaration of Helsinki and its later amendments.

### 2.2. Cell Culture

Human umbilical cord derived venous endothelial cells (HUVEC) (#C-12203, PromoCell, Heidelberg, Germany) were cultured in EGM-2 (#C-22211 and #C-39211, PromoCell, Heidelberg, Germany) at 37 °C and in a humidified atmosphere with 5% CO_2_/95% air. All other utilised cell types were maintained under the same conditions with differing media. Human dermal blood endothelial cells (HDBEC) (#C-12225, PromoCell, Heidelberg, Germany) were cultured in MV-EGM (#C-22220 and #C-39220, PromoCell, Heidelberg, Germany) and both HUVECs and HDBECs were employed in passages 3 to 6 for the experiments described here. As non-endothelial cell types, two distinct cell lines have been used, namely A549 (#ACC107, DSMZ, Braunschweig, Germany) (passages 9 to 11) and HaCaT, kindly provided by N.E. Fusenig, DKFZ, Heidelberg, Germany [23] (passages 33 to 36). While the prior epithelial cell line was cultured in RPMI (#FG1415, Biochrom, Cambridge, UK), the latter keratinocyte cell line was cultured in high glucose DMEM (#D0819, Sigma-Aldrich, St. Louis, MO, USA). Both media were supplemented with 10% (*v*/*v*) FCS (#S0115, Biochrom, Cambridge, UK) and 1% (*v*/*v*) penicillin/streptomycin (#15140122, ThermoFisher, Waltham, MA, USA). After reaching 80–90% confluency, cells were washed with DPBS (#14190144, ThermoFisher, Waltham, MA, USA) detached using Trypsin-EDTA (#25300062, ThermoFisher, Waltham, MA, USA) or StemPro^®^ Accutase^®^ solution (#A1110501, ThermoFisher, Waltham, MA, USA). Subsequently cells were either re-seeded for expansion or for the experiments. Unless stated otherwise, medium was exchanged every 2 days. 

### 2.3. Cell-Based ELISA

Serum AECA binding to ECs or other cell types in vitro was assessed in a cell-based ELISA. For this, cells were seeded at 1 × 10^4^ cells/well in transparent flat-bottom 96-well plates (#3628, Corning, Corning, NY, USA) pre-coated with a 0.2% gelatine (#G1890, Sigma-Aldrich, St. Louis, MO, USA)/DPBS solution. After reaching confluency, cells were fixed with 0.1% (*v*/*v*) glutaraldehyde (G7776, Sigma-Aldrich, St. Louis, MO, USA) for 5 min at 4 °C and subsequently washed thrice with DPBS. To avoid unspecific binding, cells were treated with 2% (*w*/*v*) BSA in DPBS, i.e., blocking buffer, for 1 h at 37 °C before addition of 0.5% (*v*/*v*) patient or control serum diluted in blocking buffer to the cells for another 1 h at 37 °C. Following serum incubation washing was performed as described above and either the horse radish peroxidase (HRP) conjugated F(ab′)2 specific anti-human IgG or anti-human IgM monoclonal antibody (#309-036-003 and #309-036-043, Jackson ImmunoResearch, West Grove, PA, USA) was incubated with the cells at 1:5000 diluted in blocking buffer for 1 h at 37 °C. Lastly, preceded by another series of washes, 3,3′, 5,5′-tetramethylbenzidine (TMB) (#34028, ThermoFisher, Waltham, MA, USA) served as a substrate for HRP and the colour reaction was stopped after 15 min using 1 M H_2_SO_4_ (#4623, Carl Roth, Karlsruhe, Germany). Optical density (OD) was measured at 450 nm using a SpectraMax^®^ microplate reader (Molecular Devices, San Jose, CA, USA). Each sample was measured in triplicates and the average absorbance was used to calculate the ELISA ratio (*ER*) as follows:

(1) (ER)=((S−A)/(B−A)) × 100,

where *S* is the OD at 450 nm of the sample while *A* and *B* indicate absorbance of the negative and positive controls, respectively. Negative control refers to cells incubated with no serum but the secondary antibody. The positive control, corresponding to an *ER* of 100, represents a SSc patient serum sample with known presence of autoantibodies against EC and non-EC targets, kindly provided by Prof. Dr. Riemekasten (Department of Rheumatology and Clinical Immunology, University of Lübeck). 

### 2.4. Endothelial Cell Small Molecule Release Assay

To examine any alterations in the HUVEC secretion profile following serum treatment, the cells were seeded in transparent flat-bottom 96-well microplates (#3997, Corning, Corning, NY, USA) at 8 × 10^3^ cells/well. Prior to the experiments, all cells were starved for 12 h in serum-free EGM-2. Next, cells were blocked with serum-free EGM-2 containing 2% (*w*/*v*) BSA for 1 h at 37 °C before incubation with 2% (*v*/*v*) patient or healthy control sera diluted in serum-free EGM-2 for 6 h. Following the serum-treatment, medium was exchanged with fresh EGM-2 to avoid any bias stemming from serum cytokines. Release of molecules into the supernatant by the serum-treated HUVEC was allowed to occur for 36 h before the supernatant was collected and debris was removed by centrifugation (1000× *g*, 2 min). Levels of myoglobin, myeloid-related protein 8/14 (MRP8/14), lipocalin A (NGAL), C-reactive protein (CRP), matrix metalloproteinase 2 (MMP-2), osteopontin (OPN), myeloperoxidase (MPO), serum amyloid (SAA), insulin-like growth factor binding protein 4 (IGFBP-4), intercellular adhesion molecule 1 (ICAM-1), vascular cell adhesion molecule (VCAM-1), MMP-9 and cystatin C in the cell supernatants were measured by LEGENDplex^TM^ Human Vascular Inflammation Panel 1 (#740551, BioLegend, San Diego, CA, USA), according to the manufacturer’s instructions. Measurements were taken on a Cytoflex LX (Beckman Coulter, Brea, CA, USA) and analysed using the LEGENDPlex^TM^ software version 8.0 (BioLegend, San Diego, CA, USA). All samples were measured in biological triplicates.

### 2.5. Flow Cytometric Detection of Endothelial Cell Activation Markers

For analysis of surface marker expression following serum treatment, HUVECs were seeded in transparent flat-bottom 48-well plates (#3548, Corning, Corning, NY, USA) at 3 × 10^4^ cells/well. Prior to the experiments, all cells were starved for 12 h in serum-free EGM-2. Thereafter, cells were blocked with serum-free EGM-2 containing 2% BSA for 1 h before incubation with 2% (*v*/*v*) patient or healthy control sera diluted in serum-free EGM-2 for 6 h. Following the serum incubation, cells were detached as described above and single cell suspensions were washed with DPBS containing 0.1% (*w*/*v*) NaN_3_ (#71289, Sigma-Aldrich, St. Louis, MO, USA) (300× *g*, 10 min). Staining was performed for 30 min at 4 °C in the dark using two separate antibody panels including either anti-human CD31(PECAM-1)-APCCy7 (#303120, BioLegend, 1:1000, San Diego, CA, USA), CD54(ICAM-1)-FITC (#116105, BioLegend, 1:100, San Diego, CA, USA) and CD62E(E-Selectin)-PE (#12-0627-42, eBiosciences, 1:100, San Diego, CA, USA) or CD106(VCAM-1)-PE (#698202, BioLegend, 1:50, San Diego, CA, USA). Additionally, a live/dead marker (#L23105, ThermoFisher, 1:50, Waltham, MA, USA) was included in both panels. To remove excess antibodies, the single cells were washed one more time as detailed above. Measurements were taken on a CytoFLEX LX (Beckman Coulter, Brea, CA, USA) and data analysis was completed using FlowJo^®^ (BD Biosciences, Franklin Lakes, NJ, USA). During gating as shown in Figure S3a–c, FSC-A low and SSC-A high cellular debris and dead cells were excluded as well as doublets by FSC-A and FSC-W plotting. To further eliminate dead cells, live/dead marker fluorescence intensity (V510) was used for gating. For analysis of surface marker expression, cellular staining was compared to n-1 controls and untreated cells. HUVECs treated with a combination of IL-1β and TNF-α at 10 ng/mL each for 6 h (#130-093-898; #130-094-014, Miltenyi Biotec, Bergisch Gladbach, Germany) were employed as a positive control. 

### 2.6. Serum Cytokine/Chemokine Measurement

Serum levels of soluble somatostatin receptor (sST2), soluble receptor for advanced glycation end products (sRAGE), sCD40L, soluble vascular endothelial growth factor receptor 1 (sVEGFR1), tumour necrosis factor superfamily member 14 (TNFSF14), TNF-α, placental growth factor (PIGF), interleukin 16 (IL)-16, IL-18, IL-10 and monocyte chemoattractant protein-1 (MCP-1) were measured by LEGENDplex^TM^ Human Vascular Inflammation Panel 2 (#740965, BioLegend, San Diego, CA, USA), according to the manufacturer’s instructions. Measurements were taken on a Cytoflex LX (Beckman Coulter) and analysed using the LEGENDPlex^TM^ software (BioLegend, San Diego, CA, USA). Additionally, levels of transforming growth factor β1 (TGF-β1) and VEGF in the serum samples were assessed using the LEGEND MAX^TM^ Total TGF-β1 ELISA kit (#436707, BioLegend, San Diego, CA, USA) and the VEGF ELISA kit (#DVE00, R&D Systems, Minneapolis, MN, USA), respectively, adhering to the manufacturer’s protocol. OD was measured using a SpectraMax^®^ microplate reader and standards prepared according to the protocol in order to determine analyte concentrations in pg/mL by 4-parameter logistic curve fitting. All samples were measured in triplicates, except for VEGF.

### 2.7. Tube Formation Assay

To analyse whether patient serum modulates angiogenesis, an in vitro Matrigel tube formation assay with HUVECs cultured in EGM-2 (passages 2–3) was performed. Cells were passaged one day before the assay (5 × 10^4^ cells on 25 cm^2^). For the assay, 1 mL aliquots of Matrigel (#354234, Corning) were thawed at 4 °C for 1 h. Tips and 48-well plates (#3548, Corning) were pre-cooled at −20 °C for 30 min. Subsequently, 130 µL of Matrigel were gently distributed into 48-wells on ice and the plate was incubated for 30 min at 37 °C and 5% CO_2_. Meanwhile, HUVECs were harvested using Trypsin-EDTA, centrifuged at 200× *g* for 5 min and seeded at 4 × 10^4^ cells/well with 5% (*v*/*v*) patient or healthy control sera diluted in EBM (#C-22210, Promocell) in a final volume of 200 µL. HUVEC treatment with a mixture of VEGF and bFGF (35 ng/mL each) or solely EBM (PromoCell) served as positive and negative controls, respectively. After 16 h incubation, five random brightfield images were taken on an AxioObserver microscope running ZEN3.4 software (Carl Zeiss Microscopy, Jena, Germany) to quantify tubular networks by performing analysis with the Angiogenesis Analyzer plugin of ImageJ 1.50i (Wayne Rasband NIH, Bethesda, MD, USA). 

### 2.8. Statistical Analysis

GraphPad Prism Version 6.0 was used for statistical analysis. Distribution of the data was tested for normality using the Shapiro-Wilks test, and not normally distributed data were analysed using non-parametric tests. For comparative analysis of quantitative parameters, the Kruska-Wallis test or the Mann-Whitney U rank-sum test were used for multiple and pairwise comparison, respectively. Correlation analysis was performed using the non-parametric Spearman coefficient. A two-tailed *p* value ≤ 0.05 was considered statistically significant. 

## 3. Results

### 3.1. Serum Factor Profile among the Patients Did Not Indicate Vascular Inflammation

A number of previous studies have focussed on dysregulated pro-inflammatory serum cytokines in ME/CFS with only partially overlapping findings underlining the often described patient heterogeneity [24,25,26]. Similarly, PCS has been associated with low-grade systemic inflammation and the vascular endothelium represents a prime target for serum inflammatory mediators [27,28]. On this note, small molecules and cytokines associated with vascular inflammation were analysed here among patient and control sera, namely: sCD40L, IL-6, IL-10, IL-18, MCP-1, PIGF, sRAGE, sST2, sVEGFR, TGF-β1, TNF-α, TNFSF14 and VEGF. 

This analysis revealed largely no differences when comparing patients (PCS and PCS/CFS) to HC individuals in terms of serum cytokine concentrations (Table 2).

Only the soluble VEGF receptor (sVEGFR) was found to be significantly reduced among both PCS and PCS/CFS patients. Despite the decreased concentrations of this decoy receptor, no significant differences in the serum levels of free VEGF could be detected here (Table 2). Other than sVEGFR, the pro-inflammatory cytokine IL-18 was found to be similarly decreased but exclusively among the PCS patients without ME/CFS vs. the HC group. Comparison between the two patient groups did not reveal any significant differences in serum cytokine concentrations.

### 3.2. Serum AECA Detection Revealed Elevated Levels among PCS/CFS Patients

**Figure 1:**
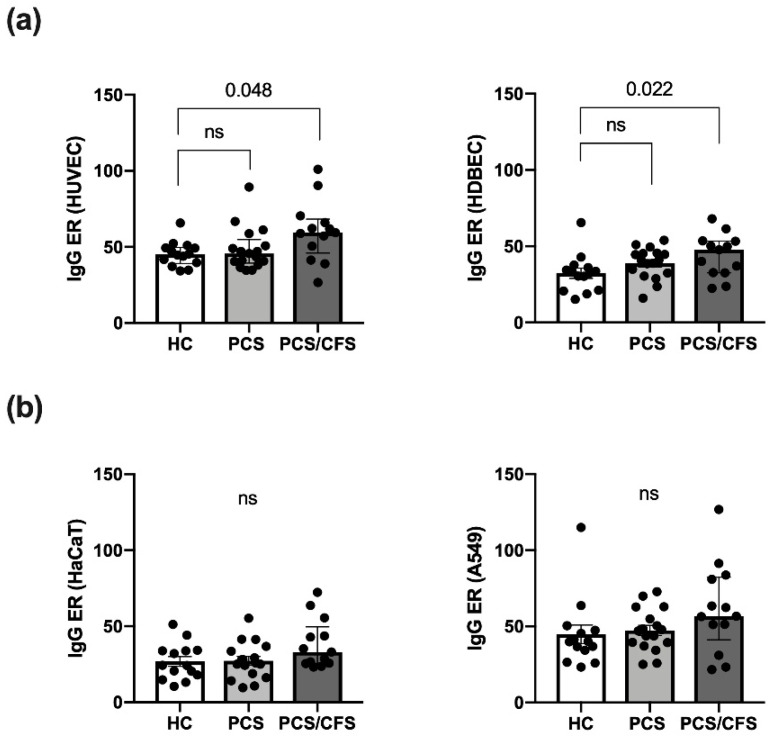
Quantitative analysis of autoantibody binding to cultured human endothelial and non-endothelial cells as determined by cell-based ELISA. Binding of autoantibodies to distinct cultured human cells was evaluated by a cell-based ELISA. Briefly, cells were incubated with 0.5% (*v*/*v*) patient (PCS, PCS/CFS) or control (HC) serum and an anti-human IgG HRP-labelled secondary antibody was used for detection of bound IgG. Shown are the IgG autoantibody levels as ELISA ratios (ER) normalised to included positive and negative controls for (**a**) two endothelial cell types i.e., macrovascular HUVEC (left) and microvascular HDBEC (right) and (**b**) two non-endothelial control cell lines, i.e., keratinocyte cell line HaCaT (left) and lung epithelial cancer cell line A549 (right). Median, interquartile range and single ER values are plotted. Statistical testing was performed using a Kruska-Wallis test. HC, healthy controls (n = 14). PCS, post-COVID-19 syndrome (n = 17). PCS/CFS, post-COVID-19 syndrome with ME/CFS (n = 13). ns, not significant (*p* value > 0.05).

Serum autoantibody binding across the patient (PCS, PCS/CFS) and HC groups was assessed here in an in vitro cell-based ELISA approach using distinct cellular substrates including the two primary ECs, i.e., macrovascular HUVEC and microvascular HDBEC. Regardless of the EC type, PCS/CFS sera displayed an overall significantly enhanced level of IgG autoantibody binding compared to the HC group, as shown by the increased ELISA ratio (ER) values (Figure 1a). At the same time no comparable differences were observed between the two patient groups. Furthermore, in contrast to IgG antibodies, no enhanced binding to HUVECs by serum IgM autoantibodies was detected (Figure S1c).

In order to rule out any bias stemming from elevated total serum IgG levels, AECA binding to HUVECs was correlated to serum IgG concentrations (Figure S1a). However, total IgG levels appeared to be comparable across the groups and there was no correlation between AECA binding to HUVECs, or HDBECs (data not shown), and IgG concentrations (Figure S1b). To further delineate whether the enhanced serum IgG binding, among PCS/CFS patient samples, was specific to ECs or the result of a broad autoreactivity towards human cells, serum IgG binding to a keratinocyte cell line (HaCaT) and an epithelial lung cancer cell line (A549) was assessed. Neither of the two non-EC cell types displayed significant differences in ER values among the patient and control groups (Figure 1b) but overall IgG reactivity appeared to be higher in PCS/CFS patients by trend.

Next, we were interested in finding out whether the enhanced levels of AECAs mediate any functional effects on ECs. While those effects can be as diverse as the AECA target structures, a commonly described feature is complement-dependent cytotoxicity (CDC) via the classical pathway [29,30]. However, in this study, dual analysis of complement component C3b deposition and subsequent lysis of HUVECs did not reveal an enhanced CDC mediated by PCS/CFS vs. HC serum AECAs. Instead, lysis was even found to be reduced by PCS/CFS serum despite no differences in C3b deposition (Figure S2b).

### 3.3. Reduced Activation Marker Surface Expression on HUVEC Following PCS/CFS and in Parts PCS Serum Incubation

Cultured HUVEC were exposed to patient or control serum (2%; 6 h) in order to examine effects on quiescent ECs and potential activation thereof. For this purpose, expression of the surface markers E-Selectin, VCAM-1 and ICAM-1 was analysed here. All of these molecules are known to be induced on activated ECs [31]. Accordingly, expression of the adhesion molecules VCAM-1, ICAM-1 and E-Selectin was enhanced on HUVECs treated with pro-inflammatory cytokines (IL-1β + TNF-α) as shown exemplarily for VCAM-1 (Figure S3d). In contrast, PECAM-1 which is involved in inter-endothelial adhesion and barrier integrity, was downregulated over time on IL-1β/TNF-α stimulated HUVECs (data not shown) as reported previously for activated ECs [32].

**Figure 2:**
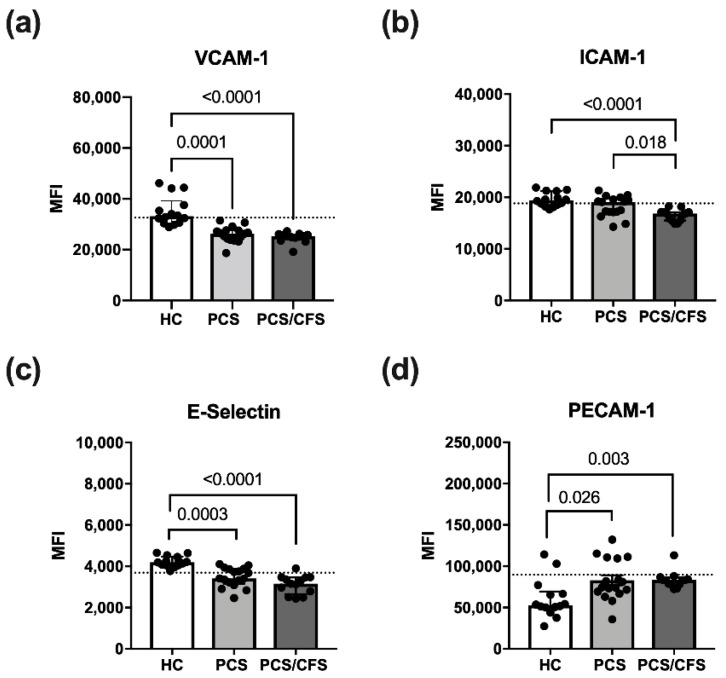
**Endothelial cell activation surface marker expression following serum incubation.** Expression levels of selected surface markers on HUVECs assessed by flow cytometry. Briefly, cultured cells were treated with 2% (*v*/*v*) patient (PCS, PCS/CFS) or control (HC) serum for 6 h before harvest and staining for flow cytometry. Examined are surface markers indicative for endothelial cell activation VCAM-1 (**a**), ICAM-1 (**b**), E-Selectin (**c**) and PECAM-1 (**d**). The expression levels are shown as mean fluorescence intensities (MFI). Dotted lines denote baseline marker expression level of untreated HUVECs. Statistical significance was determined using a Kruska-Wallis test. A *p* value ≤ 0.05 was considered significant. HC, healthy controls (n = 14). PCS, post-COVID-19 syndrome (n = 17). PCS/CFS, post-COVID-19 syndrome with ME/CFS (n = 13).

Although treatment with patient serum altered the HUVEC surface marker expression profile, it did not induce an activated EC phenotype as described above. Instead, PCS and PCS/CFS serum both led to a significant reduction in VCAM-1 and E-selectin surface expression as opposed to HC serum samples (Figure 2a,c). Moreover, surface levels of ICAM-1 were similarly reduced, but exclusively on PCS/CFS serum-treated HUVECs in contrast to both other groups (Figure 2b). In line with those observations, PECAM-1 surface expression after patient serum-treatment did not correspond to an activated EC phenotype which is characterised by its downregulation. Instead, both PCS and PCS/CFS serum enhanced PECAM-1 levels on HUVECs as opposed to HC sera (Figure 2d). Collectively, neither PCS nor PCS/CFS serum induced an activated EC phenotype in vitro. However, alternative serum-mediated effects could be observed regarding the expression of selected activation markers which significantly differed from HC serum-treated HUVECs. Those observations were largely comparable between both patient groups, except for the PCS/CFS serum-specific downregulation of ICAM-1.

### 3.4. Altered HUVEC Secretion Profile Differs between PCS and PCS/CFS Serum Incubation

In addition to analysis of surface molecules, we sought to determine changes in the HUVEC secretion profile after incubation with patient or control sera (2%; 6 h). The fine-tuned EC secretome is crucially involved in endothelial function and dysfunction due to the diversity of molecules released and produced by ECs, ranging from vasoactive mediators to pro-inflammatory cytokines or growth factors [33]. Here our focus lay on molecules whose release is implicated in vascular inflammation, i.e., MRP8/14, NGAL, CRP, MMP-2, OPN, MPO, SAA, IGFBP-4, ICAM-1, VCAM-1, MMP-9 and cystatin C, using the LEGENDPlex^TM^ Vascular Inflammation panel 1 for their detection. 

**Figure 3:**
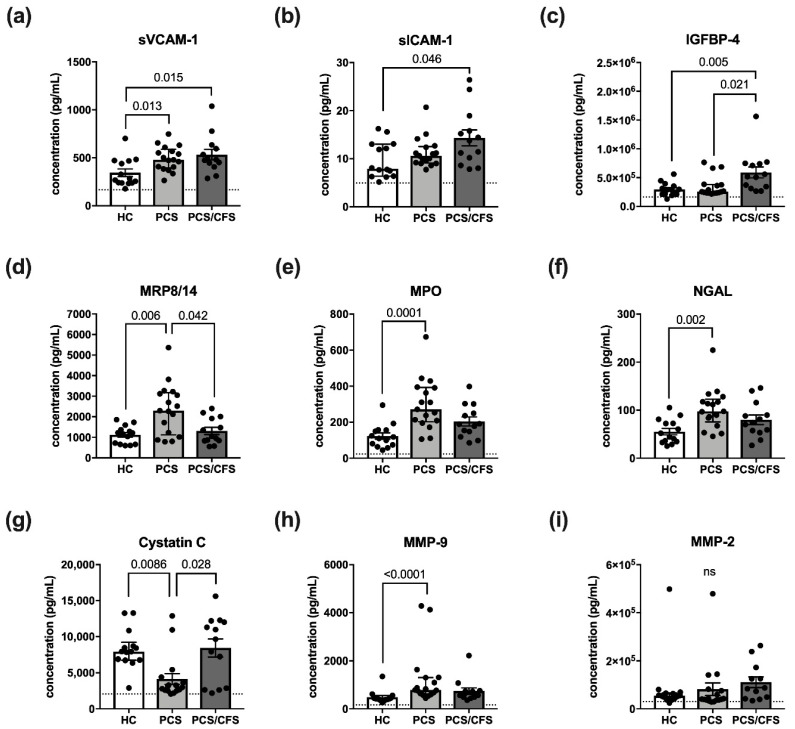
Vascular inflammation associated small molecules and their release by serum-treated cultured human endothelial cells. Briefly, cultured HUVECs were treated with 2% (*v*/*v*) patient (PCS, PCS/CFS) or control (HC) serum for 6 h. After a medium exchange, secretion was allowed to occur for 36 h. Shown are the results of a multiplex analysis using LEGENDPlex^TM^ (BioLegend) vascular inflammation panel 1 to determine the concentrations (pg/mL) of (**a**) sVCAM-1, (**b**) sICAM-1, (**c**) IGFBP-4, (**d**) MRP8/14, (**e**) MPO, (**f**) NGAL, (**g**) Cystatin C, (**h**) MMP-9 and (**i**) MMP-2. Dotted lines denote baseline level of untreated HUVEC, absence of the line indicates levels below the lower detection limits. Shown are median, interquartile range and individual values of corresponding analyte concentrations. Statistical significance was determined using a Kruska-Wallis test. A *p* value ≤ 0.05 was considered significant. HC, healthy controls (n = 14). PCS, post-COVID-19 syndrome (n = 17). PCS/CFS, post-COVID-19 syndrome with ME/CFS (n = 13). ns, not significant (*p* value > 0.05).

Interestingly, the analysed panel included the soluble versions of VCAM-1 and ICAM-1 (sVCAM-1, sICAM-1), both of which were found to be increasingly released following patient serum-treatment (Figure 3a,b). However, with respect to the latter molecule, this observation was exclusive to PCS/CFS serum-treated HUVECs. In fact, the release profile of sVCAM-1 and sICAM-1 thus turned out to be the mirror image of their respective surface expression as shown in Figure 3a,b. Other than the effects on sVCAM-1 and sICAM-1, PCS/CFS serum similarly mediated the significantly enhanced release of IGFBP-4 in comparison to both other groups (Figure 3c). Nevertheless, the most differences in small molecule secretion by HUVECs could be observed here following PCS serum-treatment which concomitantly mediated the increased release of NGAL, MMP-9, MPO and MRP8/14 (Figure 3d–f,h). At the same time, levels of the protease inhibitor Cystatin C were found to be reduced (Figure 3g). Of the 12 analytes measured, no significant differences among the groups were found regarding the release of MMP-2 (Figure 3i), myoglobin, CRP or SAA (data not shown). Furthermore, given the divergent secretion profile of serum-treated HUVECs we reproduced the experiment with patient plasma-derived extracellular vesicles (EVs) which have been postulated to play an immunomodulatory role in ME/CFS patients [34,35]. 

However, no differences in the HUVEC secretion profile were found following incubation with PCS or PCS/CFS plasma-derived EVs as opposed to the HC group (Figure S4). Taken together, both patient group′s serum samples were capable of altering the HUVEC secretion profile in comparison to the HCs. Nevertheless, there was only a small overlap between PCS and PCS/CFS serum-mediated effects on cultured EC, i.e., sVCAM-1 being increasingly released. 

### 3.5. PCS and PCS/CFS Sera Differed in Their Pro-Angiogenic Potential In Vitro

Given the crucial role of remodelling and plasticity in vascular pathophysiology, angiogenesis is unsurprisingly known to be disturbed in ED [36]. Moreover, in well-characterised vasculopathies such as SSc, patient serum has been shown to interfere with angiogenesis as well as lymphangiogenesis [37,38]. In order to analyse the potential influence of PCS and PCS/CFS sera on the angiogenic process in vitro, we performed a tube formation assay with HUVEC seeded onto a Matrigel matrix. 

**Figure 4:**
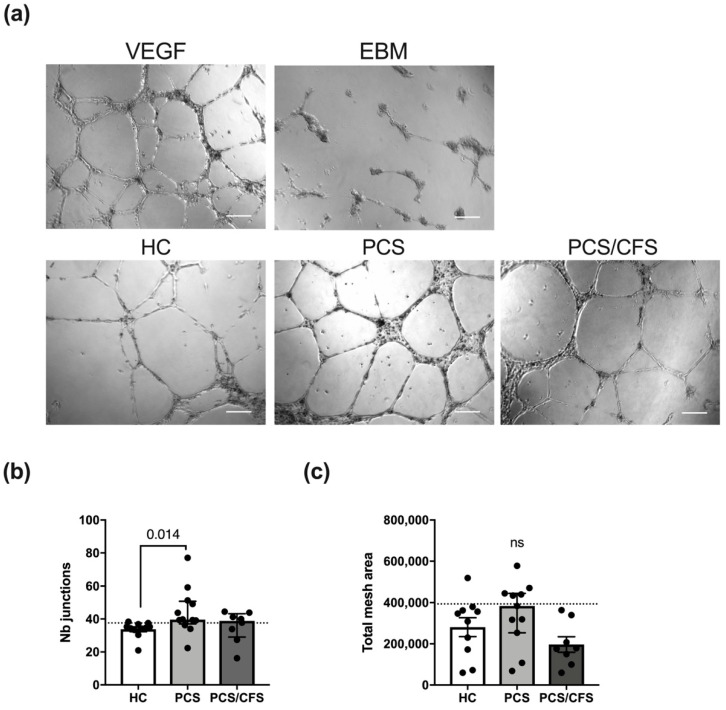
In vitro angiogenesis assay for serum-treated cultured human endothelial cells. (**a**) Representative micrographs of HUVEC treated with serum of healthy controls (HC) and both patient groups (PCS, PCS/CFS) as well as the two control settings are shown. Scale bars represent 200 µm. Experiments were performed in duplicate wells and micrographs of five areas from each well were taken. Measurements were taken with the Angiogenesis Analyzer plugin of ImageJ 1.50i. Bar graphs display the network analysis parameters (**b**) number of junctions and (**c**) total mesh area, across the patient and control groups. Shown are median, interquartile range and individual values Dotted lines denote the 90th percentile of the HC group. Statistical significance was determined using a Kruska-Wallis test. A *p* value ≤ 0.05 was considered significant. HC, healthy controls (n = 10). PCS, post-COVID-19 syndrome (n = 11). PCS/CFS, post-COVID-19 syndrome with ME/CFS (n = 8). ns, not significant (*p* value > 0.05).

The general ability to form an extensive network of interconnecting capillary-like tubes was demonstrated following HUVEC stimulation with a mixture of recombinant VEGF/bFGF (Figure 4a). Contrarily, basal cell culture medium (EBM, PromoCell) was incapable of inducing the formation of comparable tubular structures. Interestingly, patient (PCS and PCS/CFS) serum treatment (5%; 16 h) did not interfere with angiogenesis in vitro but instead PCS serum was found to significantly enhance the formation of capillary-like junctions in comparison to HC serum (Figure 4b). Although a corresponding trend could also be observed for the total mesh area, those differences among the groups did not reach statistical significance. However, when comparing the two patient groups to the 90th percentile of the HC group clear distinctions between PCS and PCS/CFS become apparent in terms of both analysed parameters. Remarkably, eight out of eleven PCS patient sera displayed a higher number of capillary junctions than the majority of the HC population, whereas the same was true only for four out of eight of the PCS/CFS patients (Figure 4b). A similar finding could be observed when looking at the total network mesh area with five out of eleven of the PCS patients showing increased levels as opposed to none of the PCS/CFS patients (Figure 4c). Overall, the majority of serum samples from PCS patients thus clearly promoted angiogenesis in vitro, in contrast to the PCS/CFS patients.

## 4. Discussion

In the context of cerebral and muscular hypoperfusion, ED is now considered to play a key role in ME/CFS pathology [7,12,13,14,39]. Moreover, ED also represents a common feature among PCS and PCS/CFS patients examined here, as reported by Haffke and colleagues [15]. The overall symptomatic resemblance between the two patient cohorts offered the unique opportunity to compare ME/CFS to another fatiguing disorder, i.e., PCS, both of which developed as post-infectious sequelae to mild or moderate COVID-19. In addition to the ability of SARS-CoV-2 to target ECs, the initial infection has the potential to elicit autoimmune reactions which have previously been proposed to play a role in ME/CFS [5]. To advance our understanding of progression to ED, we used an in vitro approach to analyse the effect of PCS and PCS/CFS sera on EC function in the present study. Despite the inherent limitations of in vitro systems, they could in fact provide many novel insights into the underlying cellular mechanisms of ED already shown in previous studies [40,41,42,43,44]. 

At the interface between the circulatory system and surrounding tissues, ECs represent a readily accessible target for a plethora of cellular as well as non-cellular components and their damage or activation has been frequently reported in chronic autoimmune diseases with the associated clinical phenotypes of vasculitis, ED or both [29,45,46,47,48]. Focussing on potential pathomechanisms related to ED, we first analysed serum for autoantibodies and cytokines, both of which have been associated with ME/CFS and ED. Overall the majority of serum cytokines analysed here did not appear to be dysregulated in our PCS and PCS/CFS patient cohorts (Table 2). Although the selected molecules only give a small glimpse at potentially dysregulated systemic molecules, a number of them have been previously associated with ME/CFS, such as IL-6, TNF-α, TGF-β1, IL-10 or VEGF [25]. Particularly, TGF-β1 could have served as a prime suspect for the observed adhesion molecule downregulation due to previous reports describing those effects on ECs in vitro [49,50,51]. However, in general there is little overlap in systemically dysregulated factors among distinct ME/CFS study populations, highlighting the frequently described patient cohort heterogeneity. Interestingly, downregulation of sVEGFR, a decoy receptor for VEGF, has been previously described in hypoxic conditions [52] and may result from a disturbed microcirculation in PCS and PCS/CFS patients. Nevertheless, serum levels of free VEGF did not differ among the groups here. 

Following recovery from COVID-19, persistent immune activation has been implicated in endothelial damage [53]. However, the pro-inflammatory cytokines analysed here did not appear to play a role in our patient cohort. In fact, IL-18 was even found to be reduced among PCS patients. Although other pro-inflammatory cytokines have not been assessed, the lack of EC activation following serum treatment, does not support the notion of cytokine-driven endothelial damage in our patient cohorts. Nevertheless, other than cytokines, AECAs were indeed elevated among PCS/CFS but not PCS patients without ME/CFS (Figure 1), indicative for an ME/CFS rather than COVID-19 specific effect. Despite the known presence of autoantibodies in ME/CFS [5], this is the first time to our knowledge that their direct binding to cultured ECs has been demonstrated. Although autoreactive IgG binding to non-EC cells types did not reach statistical significance here, it should be noted that by trend, autoantibody binding was similarly enhanced among PCS/CFS patients. Nevertheless, in the context of ED and biological significance of serum antibodies, autoantibody binding to ECs is of particular relevance here but a potentially broad autoreactivity cannot be excluded. While serum AECAs did not mediate CDC nor EC activation, antibody-dependent cellular cytotoxicity has not been evaluated here, albeit it represents another mechanism by which autoantibodies can mediate damage [45]. However, given the clinical phenotype of the patients, who did not suffer from an acute vasculitis, direct damage or cytotoxicity caused by AECAs appears to be unlikely. Any functional effects of autoantibodies in ME/CFS, i.e., here PCS/CFS, patients could be envisioned to be rather specific than damaging, as for example hypothesised for anti-G protein coupled receptor antibodies interfering with vasoregulation [39,54].

As opposed to a pro-adhesive and activated EC phenotype, patient serum reduced surface expression of the adhesion molecules VCAM-1, ICAM-1 and E-selectin (Figure 1). Given their concomitant enhanced release from cultured ECs, it can be speculated that patient sera mediated VCAM-1 and ICAM-1 shedding, whereas the latter molecule was only affected by PCS/CFS serum. In terms of plausible functional consequences, both sICAM-1 and sVCAM-1 have been attributed pro-angiogenic functions in vitro and in parts also in vivo [55,56,57,58]. Interestingly, the surface downregulation of adhesion molecules, including E-Selectin, has been reported to occur upon stimulation with pro-angiogenic factors in previous reports [59,60]. In line with this, PECAM-1 upregulation, as observed in our study, is not associated with EC activation but instead its inhibition has been shown to disturb tube formation by HUVEC [61]. 

Except for the differences in ICAM-1 expression, serum-mediated effects on EC surface molecules appeared to be comparable between PCS and PCS/CFS patients, unlike the EC secretion profile which deviated profoundly. Intriguingly, the PCS serum-mediated effects were found to support the assumption of a putatively pro-angiogenic EC phenotype. For instance, the enhanced levels of MMP-9, a matrix-metalloprotease crucial in degrading components of the extracellular matrix (ECM), were detected. Moreover, MMP-9 has been directly implicated in EC angiogenesis during hypoxia in vitro [62,63]. At the same time, secretion of NGAL, a protein which has been described to complex with MMP-9 and hereby prevent its degradation, was found to be enhanced [64,65]. Similarly to MMP-9 and NGAL, MPO was increased by PCS sera and the enzyme has also been described to promote EC angiogenesis in vitro as well as in vivo [66,67]. However, it is unclear whether this is due to its enzymatic activity or not. Other than those functions, MPO is implicated in the consumption and depletion of nitric oxide (NO) which is crucial in vasodilation and should be considered here in the context of ED [68,69].

While the aforementioned molecules were found to be induced by PCS sera, Cystatin C was released to a lesser extent. Interestingly, Cystatin C is similarly implicated in vascular remodelling because of its role as the predominant extracellular protease inhibitor [70]. Decreased Cystatin C secretion by ECs in response to PCS serum could thus further promote protease-mediated ECM degradation and facilitate angiogenesis. MRP8/14, also known as calprotectin, is mostly secreted by myeloid cells but in ECs its expression can be triggered for example by inflammatory mediators [71,72] and in the present study PCS serum could similarly promote its release (Figure 3d). While the purpose of MRP8/14 release by ECs is not entirely understood, several members of the S100 family to which MRP8/14 belongs have been described to be involved in neovascularisation [73]. On this note, low concentrations of MRP8/14 were in fact shown to promote HUVEC angiogenesis [74]. 

Strikingly, MMP-9, NGAL, MPO, MRP8/14 nor Cystatin C were found to differ following PCS/CFS serum-treatment. Instead, IGFBP-4 release was enhanced by PCS/CFS sera which has been linked to anti-angiogenic effects both in an IGF-dependent and IGF-independent manner [75,76]. The latter mechanism has been shown to be in parts due to a direct inhibition of Cathepsin B, a proteolytic enzyme involved in ECM degradation [76]. Similarly to MPO, IGFBP-4 may also be implicated in diminished NO-mediated vasodilation via inhibition of IGF-1 which plays a role in regulating endothelial NO bioavailability [77,78]. Interestingly, a recent study on ME/CFS found NO production to be reduced following HUVEC exposure to patient plasma [42].

Given our results and these considerations, we also examined the pro- or anti-angiogenic potential of patient sera and indeed this analysis revealed that PCS sera improved vascular tube formation compared to HC sera (Figure 4). In contrast, PCS/CFS serum did not promote angiogenesis which corresponds to the divergent secretion profile. Unfortunately, based on our results we cannot pinpoint any causal factor or combination of those responsible for the observed effects which clearly warrants for further examination. Nevertheless, we can rule out certain molecules such as VEGF or other serum constituents such as EVs. The latter have recently received increasing attention in ME/CFS research as potential biomarkers and also in association with immune dysregulation in the patients [35]. However, treatment with plasma-derived EVs isolated from PCS and PCS/CFS patients did not trigger any alterations in the EC secretion profile as opposed to patient serum (Figure 3c,f and Figure S4). 

While it is unlikely that the observed serum-mediated effects on EC angiogenic potential directly contribute to ED in the patients, we hypothesise a compensatory response to a disturbed microcirculation which appears to differ in PCS versus PCS/CFS. Compensatory angiogenesis is a feature described in response to vascular impairment and hypoxia [7,37,79]. In later phases of SSc, a well-studied vasculopathy, anti-angiogenic serum factors have been reported to contribute to systemically diminished angiogenesis [37,80,81]. 

**Figure 5:**
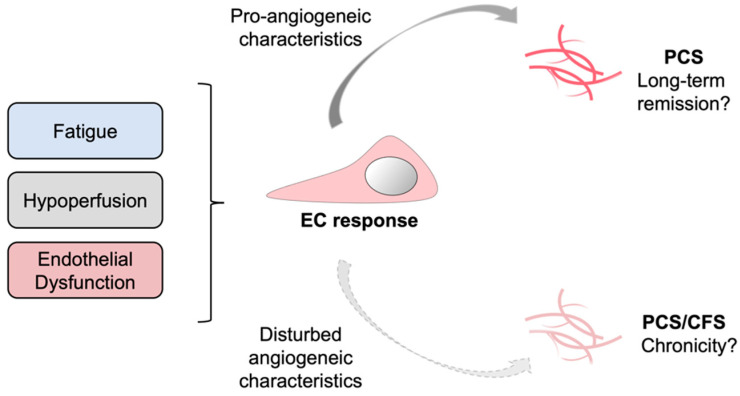
Schematic representation of a putatively suppressed angiogenic response in PCS versus PCS/CFS patients. In response to certain clinical manifestations in PCS and PCS/CFS, specifically microvascular hypoperfusion and local endothelial dysfunction, a systemic counterreaction could be triggered which directly affects endothelial cells (EC) and promotes angiogenesis. This compensatory EC response is likely to differ among PCS and PCS/CFS patients as shown in this study by a distinct secretion profile and angiogenic response of HUVECs following serum incubation. PCS, post-COVID-19 syndrome. PCS/CFS, post-COVID-19 syndrome with ME/CFS.

In our study the angiogenic potential of PCS/CFS patient sera resembled that of HCs, whereas the latter group did not suffer from ED. Contrarily, PCS patients with ED displayed a pro-angiogenic response suggesting a compensatory mechanism to ED which was absent in the PCS/CFS group. Going further, one could speculate on a contribution of lacking compensatory mechanisms to disease chronicity (Figure 5). Despite the limited number of patients in this study, the results and corresponding novel hypothesis described here could pave the way for further investigations and validation in larger cohorts. ME/CFS is known to be a long-lasting and chronic disease with a median duration of 10 years [82]. For PCS, as a recently emerged condition specific to COVID-19, there is currently no information on the disease’s time-course. However, the ongoing clinical observation of the present PCS and PCS/CFS cohorts at the Charité Fatigue Center can hopefully provide details on the disease course in the near future. 

## 5. Conclusions

In conclusion, the data presented here demonstrated PCS and PCS/CFS patient serum-mediated effects on EC function in vitro. While some functional overlap could be seen here regarding the quiescent rather than activated EC phenotypes, clear differences between both patient groups were found. Especially, the HUVEC secretion profile deviated among the patients with regards to the pro-angiogenic signature observed exclusively following PCS serum-treatment, and the distinct molecules involved in inhibition of NO, i.e., IGFBP-4 and MPO. Moreover, the pro-angiogenic release profile induced by PCS serum could be further supported by an enhanced in vitro tube formation. Based on our observations we speculate on serum factors playing a role in compensatory responses to ED and hypoperfusion in PCS, but not or insufficiently in PCS/CFS patients. Our results may thus provide a new perspective on ME/CFS chronicity which should be further examined.

## Figures and Tables

**Table 1:** Patient and control group characteristics.

	**HC (n = 14)**	**PCS (n = 17)**	**PCS/CFS (n = 13)**
**Age, Mean (Range)**	45 (31–58)	42 (27–66)	43 (24–59)
**Sex (f/m)**	(12/2)	(16/1)	(11/2)
**Months Since COVID-19 Infection, Mean (Range)**	n/a	8.3 (4.3–11.6)	9.4 (8.2–11.1)
**Bell Disability Scale, Mean (Range)**	n/a	48.24 (10–80)	45.38 (20–80)
**Chalder Fatigue Scale, Mean (Range)**	n/a	24.76 (15–32)	26 (20–33)
**PEM Score, Mean (Range)**	n/a	25.88 (17–46)	30.92 (16–44)

HC, healthy controls. PCS, post-COVID-19 syndrome. PCS/CFS, post-COVID-19 syndrome with ME/CFS. PEM, post-exertional malaise.

**Table 2:** Concentration of serum cytokines across patient and control groups.

Serum Cytokine	HC (n = 14) Median (IQR) (pg/mL)	PCS (n = 17) Median (IQR) (pg/mL)	PCS/CFS (n = 13) Median (IQR) (pg/mL)	*p* Value
sCD40L	3583 (2650–4269)	3570 (2349–4414)	3256 (2478–4219)	*p*_1_: >0.999 *p*_2_: >0.999
IL-6	6.9 (5.2–10.7)	5.9 (5.4–9.7)	6 (4.9–8.1)	*p*_1_: >0.999 *p*_2_: >0.999
IL-10	2 (1.5–3.2)	2.4 (2.1–3)	2 (1.6–2.3)	*p*_1_: 0.3500 *p*_2_: >0.999
IL-18	315.7 (263.6–341)	235.4 (167.3–260.4)	235.7 (180.6–395.5)	***p_1_: 0.0104*** *p*_2_: 0.4822
MCP-1	67.8 (55.9–100.8)	57.4 (45.1–85.8)	56.69 (46.9–67.2)	*p*_1_: 0.4611 *p*_2_: 0.4597
PIGF	10.8 (7.8–15)	10.5 (7–15.4)	11.7 (8.3–14.3)	*p*_1_: >0.999 *p*_2_: >0.999
sRAGE	410.9 (349.5–735.2)	475.8 (235.1–748.1)	345.8 (248.5–538.6)	*p*_1_: >0.999 *p*_2_: 0.6656
sST2	114.5 (71.2–213)	62.5 (42.5–196.5)	88.9 (43.3–209.1)	*p*_1_: 0.9186 *p*_2_: >0.999
sVEGFR	1983 (1728–2112)	1417 (1064–1910)	1384 (1149–1920)	***p_1_: 0.0230*** ***p_2_: 0.0500***
TGF-β1	37,529 (29,794–40,906)	41,009 (33,963–46,821)	34083 (30,939–42,905)	*p*_1_: 0.4044 *p*_2_: >0.999
TNF-α	15.35 (10.4–32.6)	24 (13.5–43.3)	23 (9.3–52.1)	*p*_1_: 0.6820 *p*_2_: >0.999
TNFSF14	36.25 (18.5–52.6)	47.53 (29.5–68.3)	42.39 (27.9–81.5)	*p*_1_: 0.5673 *p*_2_: 0.7790
VEGF	291.4 (38.20)	293.3 (38.2)	263.5 (32.24)	*p*_1_: >0.999 *p*_2_: >0.999

HC, healthy controls (n = 14). PCS, post-COVID-19 syndrome (n = 17). PCS/CFS, post-COVID-19 syndrome with ME/CFS (n = 13). For statistical analysis a Kruska-Wallis test was performed. Shown is the median and interquartile range (IQR). A *p* value ≤ 0.05 was considered significant. *p*_1_ = PCS compared with HC; *p*_2_ = PCS/CFS compared with HC.

## Data Availability

All data generated in this study will be available upon reasonable request to the corresponding author.

## Supplementary Materials

The following supporting information can be downloaded at: https://www.mdpi.com/article/10.3390/cells11152376/s1, Methods S1: Detection of complement deposition and cell lysis; Methods S2: Plasma extracellular vesicle (EV) isolation and characterization; Figure S1: Serum IgG levels across the patient and control groups as well as correlation to IgG AECA binding to HUVEC and IgM autoantibody binding; Figure S2: Complement deposition and lysis of human endothelial cells by an HLA-ABC antibody or serum AECAs; Figure S3: Gating strategy for surface marker expression analysis on HUVECs; Figure S4: Vascular inflammation associated small molecules and their release by cultured human endothelial cells treated with plasma derived extracellular vesicles (EVs).
