# Supplementary material for: Serum of Post-COVID-19 Syndrome Patients with or without ME/CFS Differentially Affects Endothelial Cell Function In Vitro
Source: Cells. 2022 Aug 2;11(15):2376. doi: 10.3390/cells11152376 (PMC9367589; doi:10.3390/cells11152376)
Supplement: Supplementary file 1 [file cells-11-02376-s001.zip › cells-1831841-supplementary.pdf]

Methods S.1. Detection of complement deposition and cell lysis

Serum AECA cytotoxic potential was assessed in a dual complement (C3b) deposition and lysis assay. Cells were seeded at 3 × 10^4 cells per well in 48-well culture plates. The next day all cells were pre-stimulated with 10 ng/mL IL-1β and TNF-α (#130-093-898; #130-094-014, Miltenyi Biotec, Bergisch Gladbach, Germany). Following the overnight pre-stimulation, medium was removed and the cells were washed with DPBS prior to blocking with 2% (w/v) BSA in serum-free EGM-2 for 1 h at 37°C. Patient serum was diluted 1:50 in the same blocking solution and heat-inactivated at 56°C for 30 min to inhibit intrinsic complement activity. Afterwards HUVECs were incubated with the serum samples for 1 h at 37°C. Alternatively, cells were either supplied with an HLA-ABC (#311402; BioLegend, San Diego, CA, USA) diluted in EGM-2 or solely culture medium, serving as a positive and negative control respectively. Cells were washed once with DPBS before baby rabbit complement (#CL3441, VH bio, Gateshead, UK) in GVB2+ buffer (#G6514, Sigma-Aldrich) (1:1) was applied for 15 min at room temperature. Following the complement incubation, cells were detached as described above and single cell suspensions were washed with DPBS containing 0.1% (w/v) NaN_3 (#71289, Sigma-Aldrich) (300× g, 10 min). Staining took place for 30 min at 4°C in the dark using a C3b-FITC antibody (#846107, BioLegend). Lastly, 7-AAD (#420403, BioLegend, San Diego, CA, USA) was diluted 1:25 in DPBS containing 0.1% (w/v) NaN_3 and added to the cells for 15 min at room temperature, in order to stain for lytic cells. Hereafter cells were kept on ice and measured within 1 h using a CytoFLEX LX (Beckman Coulter, Brea, CA, USA) multiparameter flow cytometer.

Methods S.2. Plasma extracellular vesicle (EV) isolation and characterization

Blood collection and plasma processing

EVs were isolated from peripheral blood plasma of healthy donors (HC), and both patient cohorts (PCS and PCS/CFS). To prepare plasma, blood was collected into K2E EDTA tubes (#367525, BD) and centrifuged at 900× g for 15 min at room temperature. Plasma specimens were carefully collected, stored in aliquots at −80°C and were thawed immediately prior EV isolation. After thawing, the plasma was centrifuged once at 2500 × g for 10 min at room temperature and at 5000× g for 30 min at 4°C for a sufficient removal of erythrocytes, leukocytes, and platelets. The platelet-free plasma (PFP) was then filtered through a 0.22 µm PES syringe filter (#SLHP033RS, Merck, Darmstadt, Germany).

EV isolation by size-exclusion chromatography (SEC)

For the isolation of EVs by size-exclusion chromatography (SEC), 500 µl PFP were applied on top of a qEVoriginal/35nm column (#ICO-35, IZON Science, Lyon, France) and 0.5 ml fractions were collected by an automatic fraction collector (IZON Science) with 1X PBS (#70013-016, ThermoFisher, Waltham, MA, USA) as the elution buffer after 3 ml void volume. The EV-rich fractions (1-3) from two SEC runs were pooled

and concentrated using a 10 kDa Proteus X-Spinner 2.5 Ultrafiltration concentrator (#PAL-X-10-24, Protein Ark, Rotherham, UK) by centrifugation at 2000× g for 60 min.

EV characterization

To determine quality, purity and distribution of EVs in the SEC fractions firstly the particle concentrations were measured with a ZetaView ® PMX 110 instrument according to the manufacturer´s instruction (Particle Metrix, Inning am Ammersee, Germany). Then the protein concentration of the isolated and pooled EV fractions (F1-F3) was determined using a BCA protein assay (#23227, ThermoFisher, Waltham, MA, USA). Additionally, commercial Apolipoprotein (A-I and B-100) ELISA Kits (#MBS766037 and #MBS2882822, MyBioSource, San Diego, CA, USA) were used to determine the amount of lipoprotein contamination.

To verify the successful isolation of EVs, pooled EV-rich fractions were analyzed for their characteristic surface expression of the tetraspanins CD9, CD63 and CD81, as well as for 34 other exosomal markers with human MACSPlex Exosome Kit (#130-108-813, Miltenyi Biotec, Bergisch Gladbach, USA) according to the manufacturer´s instruction.

EV uptake by HUVECs

To verify that the isolated EVs were taken up by the HUVECs, the EVs were treated with the dye PKH26 (#MINI26-1KT, Sigma-Aldrich). The stained EVs were then added in different amounts (2.5 µg, 5 µg, 10 µg and 20 µg protein mass/well) to the seeded adherent cells (1×10^5 cells/well) in a 24-well plate with 1 ml EGM-2 medium (#C-22211 and #C39211, PromoCell, Heidelberg, Germany). After 6 h of incubation at 37 °C the EVs were removed. The cells were then fixed with 4% PFA (#0335.1, Carl Roth, Karlsruhe, Germany) for 10 minutes at RT. Subsequently, HUVECs were treated with Wheat Germ Agglutinin (WGA, #29022-1, Biotium, San Francisco, CA, USA) for 10 min at 37 °C and DAPI for 15 min at RT to stain their cell membrane and nucleus, respectively. The cells were washed several times with HBSS (#14175095, ThermoFisher, Waltham, MA, USA) after each step. Subsequently, the cells were examined under a fluorescence microscope (AxioObserver, Carl Zeiss Microscopy, Jena, Germany).

Small molecule release by EV-treated HUVECs

HUVECs were treated with isolated EVs from patients and healthy controls to determine their vascular inflammation protein secretion profile. Briefly, cells were seeded in flat-bottom 48-well plates (#3548, Corning, Corning, NY, USA) at 2.4×10^4 cells/mL. Cultured cells were then treated with 20 µg EV-protein in 400 µl EGM-2 supplemented with ultracentrifuged FCS for 6 h. After changing the medium to remove the EVs, the secretion of proteins was allowed to occur for 36 h before the supernatant was collected and debris was removed by centrifugation at 1000× g for 5 min. The levels of 13 different vascular inflammation proteins (Myoglobin, CRP,

sVCAM-1, sICAM-1, IGFBP-4, MRP8/14, MMP-9, MMP-2, OPN, SAA, NGAL, Cystatin C and MPO) in the supernatant were measured by LEGENDplex™ Human Vascular Inflammation Panel 1 (#740551, BioLegend, San Diego, CA, USA) according to the manufacturer´s instruction. An untreated and a PBS-treated control were included. All samples were measured in biological triplicates. Data are shown in Figure S4.

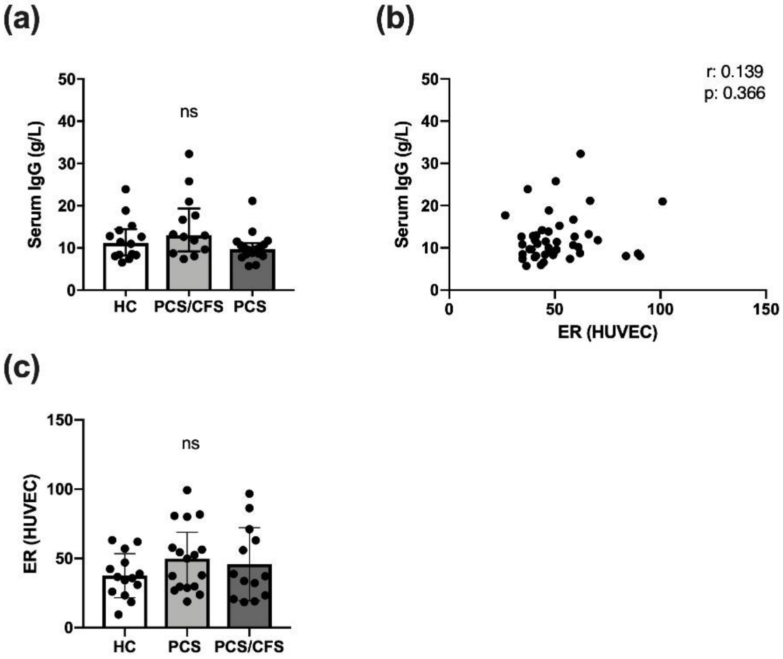

Figure S1. Serum IgG levels across the patient and control groups as well as correlation to IgG AECA binding to HUVEC and IgM autoantibody binding. (**a**) Serum IgG levels as determined by ELISA (#3850-1H-6, Mabtech, Stockholm, Sweden). (**b**) Association between total serum IgG levels (g/mL) and ELISA ratios for IgG autoantibody binding to HUVEC, determined by cell-based ELISA. Shown is Spearman's rank correlation analysis which was used to determine the correlation coefficient r and two-tailed p-value as displayed on the graph. **(c)** Binding of IgM autoantibodies to HUVEC cells was evaluated by a cell-based ELISA. Shown are median, interquartile range and individual values. Statistical analysis using a Kruska-Wallis test. A p value ≤ 0.05 was considered significant. HC, Healthy controls (n=14). PCS, post-COVID-19 syndrome (n=17). PCS/CFS, post-COVID-19 syndrome with ME/CFS (n=13). Ns, not significant.

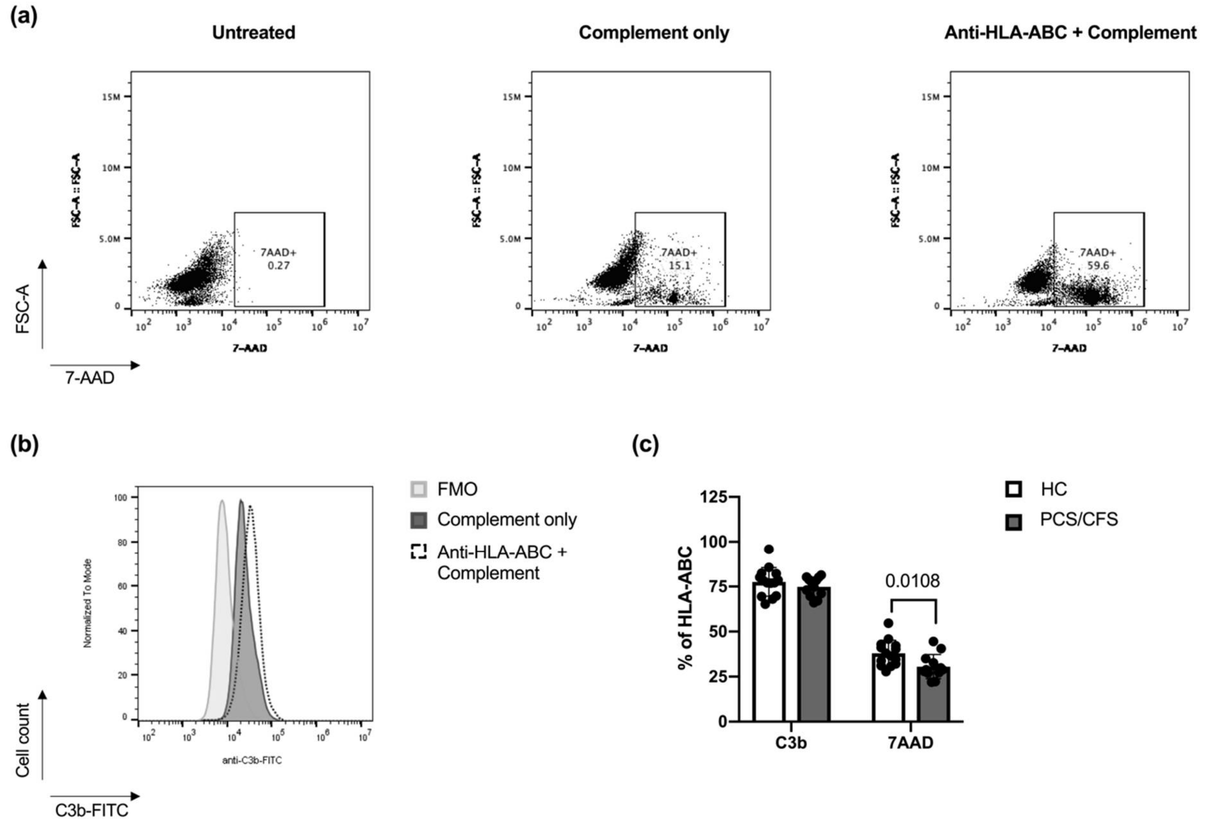

Figure S2. Complement deposition and lysis of human endothelial cells by an HLA-ABC antibody or serum AECAs. Dual assessment of C3b deposition and lytic cells (7-AAD+) by flow cytometry as a proxy for complement mediated endothelial cell damage. Briefly, HUVECs were incubated with anti-HLA-ABC, 1% (v/v) serum diluted in culture medium or left untreated for 1h. Subsequently baby rabbit complement was added for 15 min. Thereafter, combined staining with a C3b-FITC antibody and 7-AAD was performed, followed by flow cytometric measurement. (**a**) Dot plots display the 7-AAD signal intensity and percentage of 7-AAD positive cells was defined based on the unstained control. Shown are untreated control cells (left), complement only treatment (middle) and the anti-HLA-ABC antibody plus complement-treated HUVECs (right). Histogram in (**b**) shows the mean fluorescence intensity (MFI) for the C3b-FITC staining. (**c**) Levels of anti-C3b-FITC and 7-AAD staining following treatment of HUVEC with patient or control serum. The MFI of anti-C3b-FITC and the percentage of 7-AAD+ cells were normalised to the level of the anti-HLA-ABC treated control cells (% of HLA-ABC). Shown are median, interquartile range and individual values of corresponding analyte concentrations. Statistical significance was determined using a Mann-Whitney-U rank-sum test. FMO, fluorescence minus one control (n-1). HC, healthy controls (n=14). PCS/CFS, post-COVID-19 syndrome with ME/CFS (n=12).

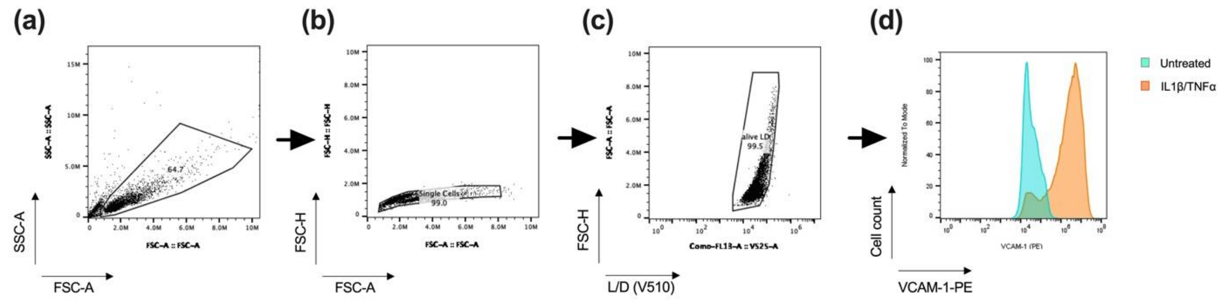

Figure S3. Gating strategy for surface marker expression analysis on HUVECs. Scatter plots show exclusion of cellular debris (**a**) and doublets (**b**). Thereafter, dead cells were excluded by staining with a live/dead (V510) marker (**c**). (**d**) Exemplary histogram is shown for the shift in VCAM-1 fluorescence intensity following HUVEC stimulation with a combination of the pro-inflammatory cytokines IL-1β and TNF-α (10 ng/mL) for 6 h, compared to untreated cells. MFI, mean fluorescence intensity.

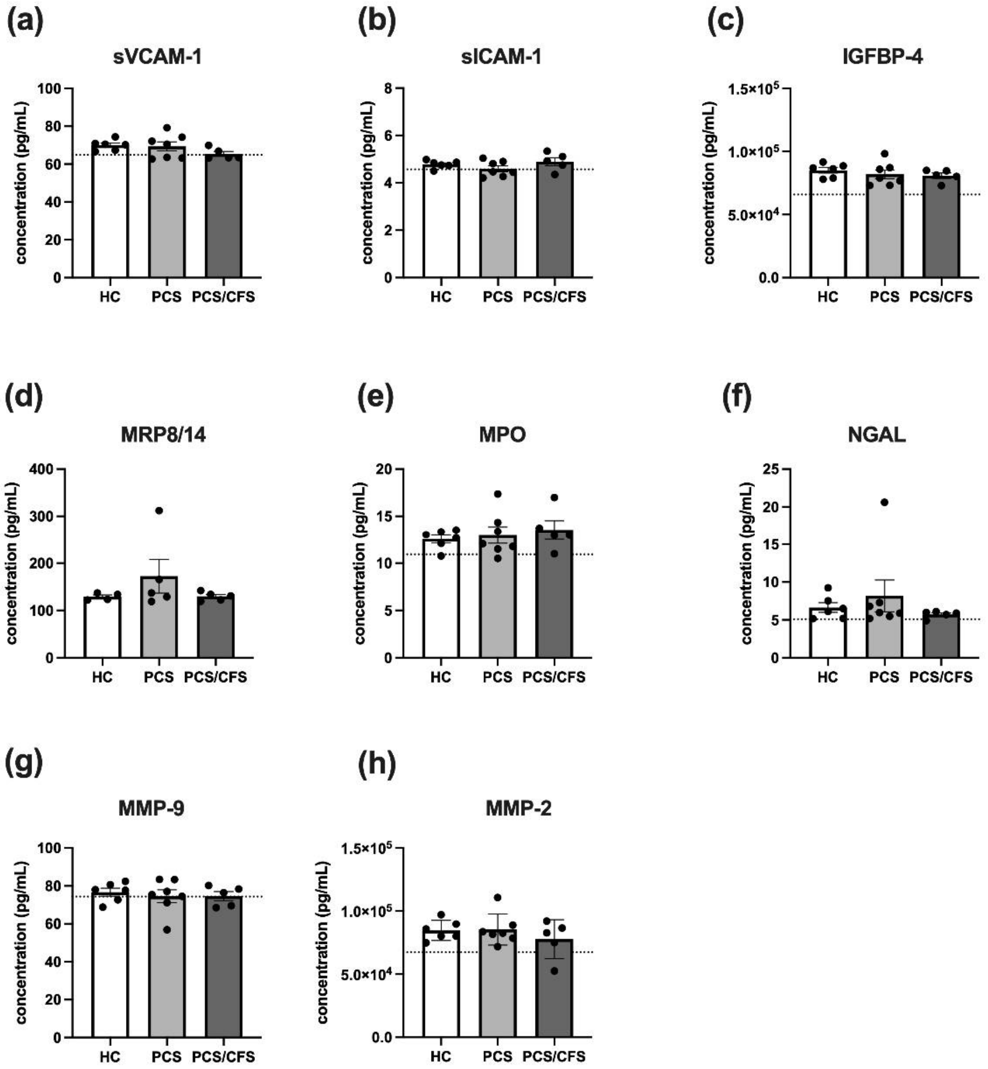

Figure S4. Vascular inflammation associated small molecules and their release by cultured human endothelial cells treated with plasma derived extracellular vesicles (Evs). Alterations in small molecule secretion profile of HUVECs following EV stimulation. Briefly, cultured cells were treated for 6 h with 5µg EV/well in PBS isolated by SEC from (PCS, PCS/CFS) or control (HC) plasma. As a control the cells were treated with PBS in equal volume as Evs. After a complete medium exchange, secretion was allowed to occur for 36 h. Shown are the results of a multiplex analysis using LEGENDPlex™ (BioLegend, San Diego, CA, USA) vascular inflammation panel 1 to determine the concentrations (pg/mL) of (**a**) sVCAM-1, (**b**) sICAM-1, (**c**) IGFBP-4, (**d**) MMP-9, (**e**) NGAL, (**f**) MRP8/14, (**g**) MPO and (**h**) MMP-2. Dotted lines denote baseline level of PBS-treated HUVEC, absence of the line indicates levels below the lower detection limits. Shown are median, interquartile range and individual values of corresponding analyte concentrations. Statistical significance was determined using a Kruska-Wallis test. A p value ≤ 0.05 was considered significant. HC, healthy controls (n=5-6). PCS, post-COVID-19 syndrome (n=7). PCS/CFS, post-COVID-19 syndrome with ME/CFS (n=5).
